# Intrauterine Nitric Oxide Deficiency Weakens Differentiation of Vascular Smooth Muscle in Newborn Rats

**DOI:** 10.3390/ijms22158003

**Published:** 2021-07-27

**Authors:** Anastasia A. Shvetsova, Anna A. Borzykh, Ekaterina K. Selivanova, Oxana O. Kiryukhina, Dina K. Gaynullina, Olga S. Tarasova

**Affiliations:** 1 Department of Human and Animal Physiology, Faculty of Biology, M.V. Lomonosov Moscow State University, 119234 Moscow, Russia; anastasiashvetsova92@gmail.com (A.A.S.); blamanche@ya.ru (E.K.S.); Dina.Gaynullina@gmail.com (D.K.G.); 2 Laboratory of Exercise Physiology, State Research Center of the Russian Federation-Institute for Biomedical Problems, Russian Academy of Sciences, 123007 Moscow, Russia; borzykh.anna@gmail.com; 3 Laboratory for the Study of Information Processes at the Cellular and Molecular Levels, Institute for Information Transmission Problems, Russian Academy of Sciences, 119333 Moscow, Russia; kcyu@yandex.ru; 4 Department of Physiology, Russian National Research Medical University, 117513 Moscow, Russia

**Keywords:** nitric oxide, preeclampsia, prenatal ontogenesis, vascular smooth muscle cells, synthetic phenotype, contractile phenotype

## Abstract

Nitric oxide (NO) deficiency during pregnancy is a key reason for preeclampsia development. Besides its important vasomotor role, NO is shown to regulate the cell transcriptome. However, the role of NO in transcriptional regulation of developing smooth muscle has never been studied before. We hypothesized that in early ontogeny, NO is important for the regulation of arterial smooth muscle-specific genes expression. Pregnant rats consumed NO-synthase inhibitor L-NAME (500 mg/L in drinking water) from gestational day 10 till delivery, which led to an increase in blood pressure, a key manifestation of preeclampsia. L-NAME reduced blood concentrations of NO metabolites in dams and their newborn pups, as well as relaxations of pup aortic rings to acetylcholine. Using qPCR, we demonstrated reduced abundances of the smooth muscle-specific myosin heavy chain isoform, α-actin, SM22α, and L-type Ca^2+^-channel mRNAs in the aorta of newborn pups from the L-NAME group compared to control pups. To conclude, the intrauterine NO deficiency weakens gene expression specific for a contractile phenotype of arterial smooth muscle in newborn offspring.

## 1. Introduction

Nitric oxide (NO) is one of the most important regulatory molecules in the cardiovascular system. NO is produced by the vascular endothelium and has a potent vasodilatory effect. There are three isoforms of the enzyme that synthesizes NO: endothelial, neuronal, and inducible NO synthases (eNOS, nNOS, and iNOS, respectively). In a healthy organism, the endothelial isoform of the enzyme predominates in the vascular endothelium [1,2].

The production of NO by the endothelium can undergo significant changes during early ontogenesis in different regions of the vascular bed. In the early postnatal period, the contribution of NO to the regulation of vascular tone is large in the gastrointestinal tract, skin, the preglomerular arterioles of the kidney, and skeletal muscle feed arteries [1,3]. Along with that, in the vascular beds of the lungs and brain, skeletal muscle arterioles, and interlobar kidney arteries, the contribution of NO to vasoregulation, on the contrary, is relatively low in the period of early ontogenesis and increases with age [1,3]. Importantly, at the systemic level, the contribution of NO to the regulation of blood pressure level is higher in the period of early postnatal ontogenesis compared to the adult organism [3]. It is likely that an integrally high level of NO production in early ontogenesis is important for maintaining low blood pressure [3,4], to reduce heart afterload and prevent damage of immature vasculature.

Along with that, it is known that NO and its main effector in vascular smooth muscle, protein kinase G (PKG) inhibit proliferation and accelerates the differentiation of cultured vascular smooth muscle cells [5,6]. It has been shown that activated PKG induces an increase in the expression of the smooth muscle isoform of the myosin heavy chain and α-actin [6,7,8], as well as the SM22α, a marker of the differentiated smooth muscle cell [9]. Such control is accomplished, at least in part, at the transcriptional level, since several studies demonstrated simultaneous stimulatory effects of NO on mRNA and protein contents of the differentiation markers [7,8]. Importantly, differentiating effects of NO have been observed under in vivo conditions. The eNOS mutant mice displayed an abnormal arterial remodeling due to a hyperplastic response of the media [10]. By contrast, either eNOS [11] or nNOS [12] gene transfer inhibited intimal hyperplasia in the injured vessel wall and modulated intimal smooth muscle cell phenotype toward a more differentiated one [12].

Therefore, vascular smooth muscle cells undergo proliferation and dedifferentiation in either cell culture or during vessel wall injury, and NO is a powerful stimulus to suppress these processes. The opposite processes occur during the development of the vascular system in embryogenesis when the conversion of smooth muscle cells from the synthetic to the contractile phenotype takes place [13]. To our knowledge, the role of NO in the control of differentiation of smooth muscle cells during early ontogenesis remains unclear. Of note, the mitogenic control mechanisms may differ in embryonic and adult vascular smooth muscle cells [14].

This work tested the hypothesis that, in early ontogenesis, the action of highly abundant NO is favorable for the expression of genes specific to the contractile phenotype of arterial smooth muscle. If so, NO deficiency during intrauterine development would attenuate the expression of smooth muscle differentiation markers. To test this, we supplemented pregnant rat females with NOS inhibitor, to suppress NO production in developing fetuses.

## 2. Results

### 2.1. Characteristics of Females

**Figure 1:**
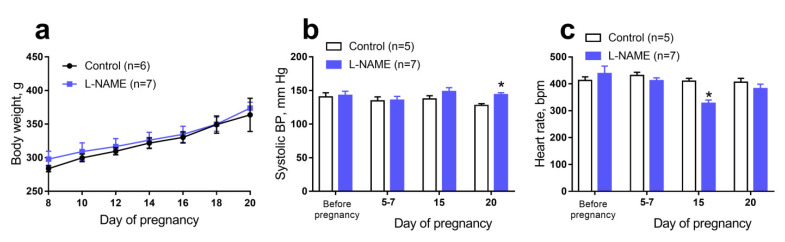
Characteristics of females from control and L-NAME groups. (**a**) Bodyweight gain during the pregnancy. (**b**,**c**) Systolic blood pressure (BP, b) and heart rate (**c**) before pregnancy, at gestational day (GD)5-7, GD15, and GD20; *n*—number of females. Data are presented as mean and S.E.M. * *p* < 0.05 (Unpaired Student’s *t*-test).

The bodyweight of females consuming L-NAME did not differ from the weight of control females throughout pregnancy (Figure 1a). Water consumption by pregnant females rose progressively during gestation but did not change in relation to the body weight, similar to that observed in our previous study [15]. The daily relative water consumption throughout the pregnancy did not differ between control and L-NAME groups: 141 ± 8 mL/kg and 146 ± 11 mL/kg, respectively (*p* > 0.05). The average daily dose of L-NAME consumed by females of the experimental group from day 10 of pregnancy to delivery was 78 mg/kg. Consumption of L-NAME led to a decrease in the content of NO metabolites in the blood serum of females on the 18–19 day of pregnancy (10 ± 3 μM (*n* = 7) in the L-NAME group and 17 ± 5 μM (*n* = 5) in the control group, *p* < 0.05).

Systolic blood pressure (BP) and heart rate did not differ in female rats of the control and L-NAME groups before pregnancy and on gestational days (GD) 5–7 (before the start of L-NAME consumption, Figure 1b,c). On GD15, the BP level did not differ between the two groups (Figure 1b) but the heart rate was lower in the females of the experimental group (Figure 1c). On GD20, females who consumed L-NAME had higher BP (Figure 1b) and unchanged heart rate in comparison with females of the control group (Figure 1c).

### 2.2. Gross Characteristics of the Offspring

The duration of gestation periods were 21 days in control and 22 days in L-NAME-treated dams. The number of pups per litter did not differ between the two groups (12 ± 1 in control and 11 ± 1 in L-NAME, *p* > 0.05). Two-thirds (66%) of the pups born to females consuming L-NAME had limb reduction defects. On the next day after birth, the offspring of the L-NAME group was characterized by reduced body weight, their relative heart weight was increased, but the relative kidney weight did not change compared to age-matched offspring of the control group (Table 1). The content of NO metabolites in pup blood serum was reduced in the L-NAME group compared to the control group (Table 1).

### 2.3. Functional Studies of the Aorta of Rat Pups

**Figure 2:**
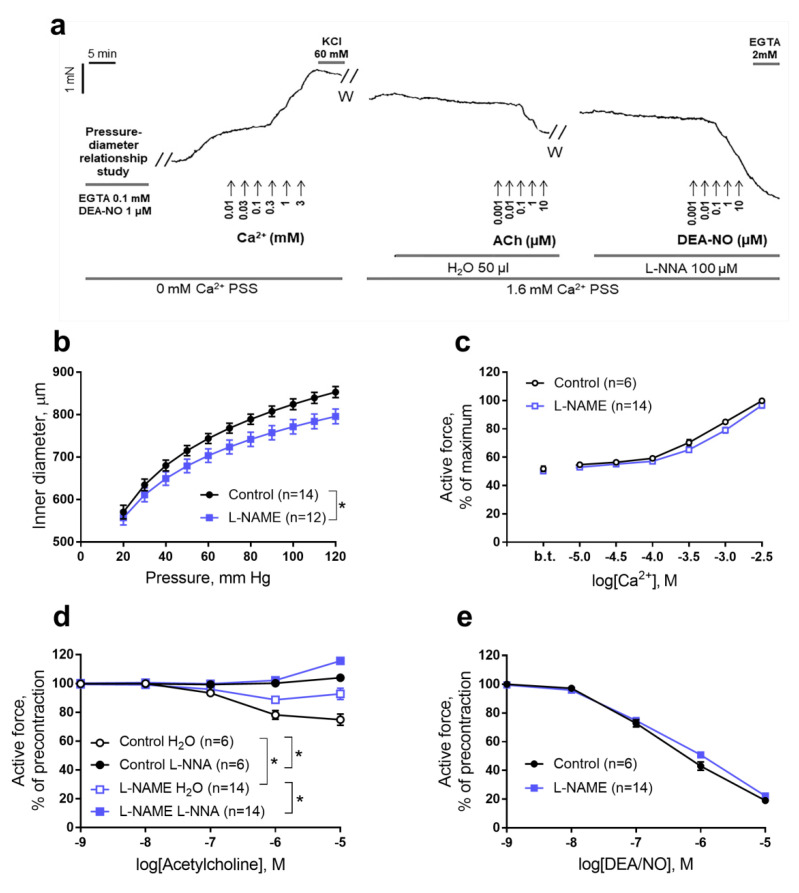
Functional studies of aortic segments of 1–2-day-old rat pups born to control and L-NAME-treated females. (**a**) Representative recording of the experiment on the aortic segment from the “Control H_2_O” group. (**b**) Passive transmural pressure-inner diameter relationships. (**c**) Aortic contractile responses to a stepwise increase of extracellular Ca2+ concentration. (**d**) The responses to acetylcholine in the presence of solvent (H2O) or NO-synthase inhibitor L-NNA (100 µM). (**e**) The responses to NO-donor DEA/NO in the presence of 100 µM NO-synthase inhibitor L-NNA. B.t.—basal tone; *n*—number of rat pups. Data are presented as mean and S.E.M. * *p* < 0.05 (Repeated Measures ANOVA). ACh—acetylcholine, PSS—physiological salt solution, W—washout.

The experiments were carried out according to the protocol shown in Figure 2a (see Materials and Methods for detailed description). The aorta of rat pups from the L-NAME group demonstrated a less steep passive pressure-diameter relationship (Figure 2b) and a reduced normalized inner diameter (d_100_, see Materials and Methods for description) compared to the aorta of control pups (Table 2). At the same time, the maximum tension developed by the aorta (defined as the ratio of the maximum force to two lengths of the aortic segment) did not differ between the groups (Table 2).

At zero Ca^2+^ concentration in the external solution aortic segments of pups from both groups developed a basal tonic contraction equal to about 50% of the maximum active force (Figure 2a,c). Stepwise increase of external Ca^2+^ concentration evoked contractile responses of the aorta that did not differ between control and L-NAME pups (Figure 2c).

Aortic segments of both pup groups showed endothelium-dependent relaxations to acetylcholine, however, the responses to acetylcholine in the L-NAME group were weaker than in the control group (Figure 2d). The latter is confirmed by calculating the area above the concentration-response curve to acetylcholine (41 ± 6 a.u. in the control (*n* = 6) and 24 ± 2 a.u. in the L-NAME group (*n* = 14), *p* <0.05). Importantly, in both groups acetylcholine-induced aortic relaxations were blocked by the NO-synthase inhibitor L-NNA (Figure 2d). In the presence of L-NNA, the responses to acetylcholine no longer differed between groups (Figure 2d). Endothelium-independent aortic relaxation to the NO donor DEA/NO did not differ between the groups (Figure 2e).

### 2.4. Comparison of mRNA Expression Levels in the Aorta of Rat Pups from the Control and L-NAME Groups

**Figure 3:**
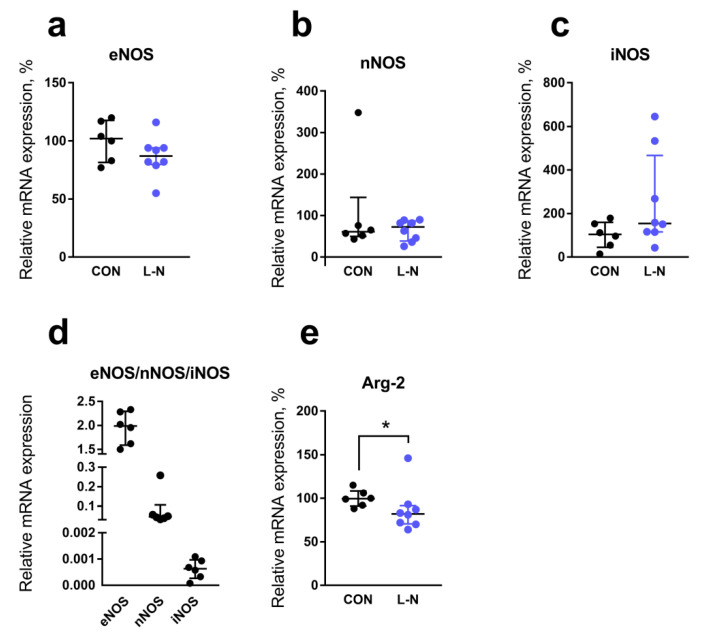
Relative mRNA expression levels of genes related to NO production in the aorta of 1-day-old rat pups. (**a**–**c**,**e**) Relative mRNA content of eNOS (**a**), nNOS (**b**), iNOS (**c**) and Arginase-2 (**e**) in aorta of control or L-NAME (L-N) rats. (**d**) Relative mRNA content of eNOS, nNOS, and iNOS in the aorta of control rat pups. The number of tissue samples per group: control—6; L-NAME—8. Data are presented as median and interquartile ranges. * *p* < 0.05 (Mann-Whitney test).

Further, we compared mRNA expression levels of genes related to NO production in the aorta of two groups. The mRNA content of endothelial, neuronal, and inducible NO synthase isoforms (eNOS, nNOS, and iNOS, respectively) did not differ between pups from control and L-NAME groups (Figure 3a–c). Of note, the main isoform expressed in the aorta of control rat pups was eNOS, while the contents of nNOS and iNOS mRNA were prominently lower (Figure 3d). The mRNA content of arginase-2, which competes with eNOS for the substrate L-arginine, was slightly reduced in the aorta of the L-NAME group (Figure 3e).

**Figure 4:**
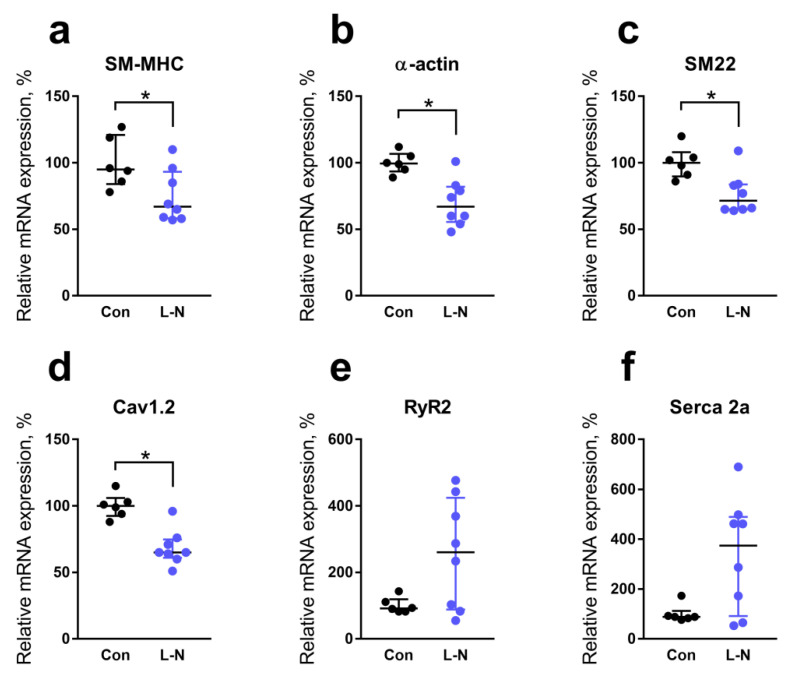
Relative mRNA expression levels of genes encoding proteins typical of the smooth muscle cell contractile phenotype in the aorta of 1-day-old rat pups. Relative mRNA content of smooth muscle myosin heavy chain isoform (SM-MHC, (**a**), α-actin (**b**), SM22α (**c**), L-type Ca^2+^ channels (Cav1.2, (**d**)), ryanodine receptor 2 (RyR2, (**e**) or Serca2a (**f**) in the aorta of control or L-NAME (L-N) rats. The number of tissue samples per group: control—6; L-NAME—8. Data are presented as median and interquartile ranges. * *p* < 0.05 (Mann-Whitney test).

As the next step, we compared the expression levels of genes that encode smooth muscle contractile and regulatory proteins and change their activity during the differentiation of smooth muscle cells (for gene names see Table 3). Relative mRNA content of the smooth muscle-specific myosin heavy chain isoform was reduced in the aorta of L-NAME rat pups compared to control animals (Figure 4a). Similarly, the contents of α-actin and SM22α mRNA were decreased in the aorta of L-NAME rat pups compared to control animals (Figure 4b,c). The mRNA content of L-type Ca^2+^ channels was also reduced in the aorta of L-NAME rat pups in comparison with the control group (Figure 4d). However, ryanodine receptor 2 (RyR2) mRNA and Serca2a mRNA contents were not reduced in the aorta of rat pups from the L-NAME group (Figure 4e,f).

## 3. Discussion

### 3.1. Maternal L-NAME Treatment Provides Fetal NO Deficiency in the Rat

L-NAME consumption by females during pregnancy led to the reduction of the NO metabolites level in their blood and, importantly, in the blood of their newborn offspring. This suggests that NO production was suppressed in the second half of prenatal development and, therefore, the used experimental model was relevant for the proposed goal of this study.

The treatment with L-NAME did not change dams’ body weight and water consumption but led to the increase of systolic BP level at the term of pregnancy due to an increase of vascular resistance with the diminished vasodilatory influence of NO. Probably, on the 15th day of pregnancy, vascular resistance in L-NAME-treated dams was increased as well, but its contribution to BP level was counterbalanced by the fall in the cardiac output, as seen from lower heart rate values. Of note, the treatment of animals with L-NAME is a commonly used model of preeclampsia [16,17]. In our model, we observed such manifestations of preeclampsia as increased blood pressure level and attenuated NO bioavailability [18,19,20]. Therefore, the findings of the present study should be considered when exploring potential consequences of preeclampsia in the vasculature of newborn offspring.

L-NAME consumption induced moderate changes in pregnant females but had a pronounced effect on their offspring. Of note, previous studies demonstrated the rapid distribution of L-NAME from maternal blood to the fetus [21]. Blood concentration of NO metabolites in our study was much higher in control newborn pups than in their mothers, which points to the important role of NO in the control of fetus development. Newborn offspring of the L-NAME group were characterized by decreased body weight and many pups from the L-NAME group had limb defects. Previous studies associated teratogenic effects of L-NAME with malformation of limb vasculature and hemorrhage [21,22]. Similar defects were observed in eNOS-deficient mice [23].

### 3.2. Maternal L-NAME Treatment Reduces NO Bioavailability in the Vasculature of Newborn Offspring

The primary aim of our study was to assess the role of NO in the development of fetal vasculature, therefore, we further focused on the cardiovascular consequences of maternal L-NAME treatment in the offspring. Endothelium-dependent relaxation of the aorta to acetylcholine was decreased in newborn pups from the L-NAME group due to the reduction of NO-mediated signaling, as we showed using an inhibitor L-NNA. Our data are supported by previous findings of another group where the reduction of endothelium-dependent dilation to acetylcholine due to impairment of NO-mediated signaling was described in aortic segments of term fetuses born to L-NAME-treated dams [24].

Importantly, reduction of NO-mediated signaling in pups born to L-NAME-treated females had an endothelial origin since the smooth muscle responsiveness to NO did not differ between groups. Lower NO production in the aortic endothelium of pups from the L-NAME group was presumably associated with reduced activity of eNOS since the abundance of eNOS mRNA (the main isoform of NO-synthase in the aorta of newborn rats) was not affected by maternal treatment with L-NAME.

The inner diameter and compliance of the aorta were reduced in pups from the L-NAME group compared to control pups. Together with increased heart weight, this might point to the elevated blood pressure in newborn pups from the L-NAME group because of impaired NO-signaling in systemic vasculature. The latter, however, needs to be proved experimentally in future studies.

### 3.3. Reduced NO Bioavailability in the Vasculature of Newborn Offspring Is Associated with Altered Smooth Muscle Cell Properties

L-NAME penetrating to the developing organism through the placenta [21] inhibited NO production in the endothelium located close to aortic smooth muscle cells. Our data show that such NO deficiency affected the process of smooth muscle cells differentiation in the aortic wall. Pups born to L-NAME-treated females had decreased mRNA contents of all studied smooth muscle differentiation markers, such as α-actin, smooth muscle-specific myosin heavy chain isoform, and SM22α [25]. Combining our results with previously obtained in vitro [6,7,8,9] and in vivo [10,11,12] data, we may conclude that the deficiency of NO-mediated PKG signaling in smooth muscle cells of developing aorta impairs the transcription of genes encoding smooth muscle differentiation markers. Therefore, aortic smooth muscle cells of newborn rat pups from the L-NAME group are less differentiated, because of NO deficiency during development.

Despite potentially reduced smooth muscle myosin content, maximal aortic contractility was not changed in the progeny of L-NAME-treated dams. However, non-muscle myosin, which is abundant in undifferentiated smooth muscle cells [25], is also responsible for smooth muscle contractility, especially for the sustained phase of the response [26]. Of note, the activity of non-muscle myosin is regulated by the Rho/Rho-kinase signaling pathway [26], with high activity being a hallmark of developing vascular smooth muscle [27].

The deficiency of endothelial NO production in early ontogenesis did not change aortic contractile responses to increasing Ca^2+^ concentration in the external solution. However, the mechanisms regulating smooth muscle cell contractility seem to be altered, since we observed the decreased expression of L-type Ca^2+^ channels along with unchanged expression of Serca2a and RyR2 in the aorta of newborn rats from the L-NAME group. Presumably, under conditions of NO-deficiency, the mechanisms of Ca^2+^ handling of arterial contractility change towards the smaller contribution of Ca^2+^ from the external space. In addition, deficiency of L-type Ca^2+^ channels in aortic smooth muscle cells of pups from the L-NAME group might be compensated by a higher influx through voltage-independent Ca^2+^ channels [28], including store-operated channels that are more characteristic of undifferentiated vascular smooth muscle cells [29].

To our knowledge, the effects of NO on the transcription of genes encoding the proteins involved in Ca^2+^-homeostasis of vascular smooth muscle cells have not been studied before. L-type Ca^2+^ channels are typical of differentiated smooth muscle cells [30]. So, the decrease of their expression in the aorta of L-NAME-pups can be considered as one more mechanism of NO-mediated phenotypic modulation of vascular smooth muscle.

One would expect that the expression of Serca2a and RyR2 in rat pups from the L-NAME group would also be reduced since they are typical of contractile smooth muscle cells [31], but it did not take place. Obviously, the development of vascular smooth muscle cells is controlled by multiple mechanisms, the contribution of which may depend on the degree of smooth muscle cell differentiation. For example, dedifferentiation of rat aortic smooth muscle cells in culture resulted in the disappearance of smooth muscle myosin heavy chain, RyR, and Serca2a mRNAs [32]. However, plating cells at high density prevented loss of Serca2a and RyR, but not myosin heavy chains, pointing to the differential control of these proteins expression in vascular smooth muscle cells. Although underlying mechanisms are not yet clear, sarcoplasmic reticulum-dependent regulation may compensate for the shortage of cell contractility control by extracellular Ca^2+^ influx in the aorta of L-NAME-pups.

Therefore, our results point to the role of NO in the control of vascular smooth muscle cell differentiation. The limitation of our study is that the observed changes in mRNA expression of differentiation markers have not been validated on the protein level, as was done in several previous studies [7,8]. Such experiments together with more mechanistic evaluation of functional consequences of prenatal NO deficiency are the topics of future studies.

## 4. Materials and Methods

Experiments were performed using Wistar rats in accordance with the European Convention on the protection of animals used for scientific purposes (EU Directive 2010/63/EU). Animal procedures were approved by the Moscow State University committee on animal welfare (protocol 133-g, approval date 08.10.2020). Sexually mature male and female Wistar rats were obtained from the Federal State Budgetary Scientific Institution of Research Institute of General Pathology and Pathophysiology Russian Academy of Sciences and then bred in the laboratory animal unit of the Biological Faculty, Moscow State University. The animals were maintained on 12 h light-dark cycle and had free access to the normal rodent chow (Laboratorkorm, Moscow, Russia).

### 4.1. Model of Fetal NO Deficiency in the Rat

Suppression of NO production in the prenatal and neonatal periods in rat pups was carried out by suppression of NO production in the organism of their mothers. For this purpose, sexually mature male and female rats were placed together overnight. The onset of pregnancy was determined by the presence of sperm in the vaginal smear the next morning (this day was considered the first GD). On GD10, females were randomly assigned to control or L-NAME groups. Females of the L-NAME group received L-NAME in drinking water (at a concentration of 500 mg/L) from GD10 until delivery, while females of the control group received tap water. During the pregnancy, the females of both groups were regularly weighed, and their water intake was recorded.

During the week before mating, female systolic BP and heart rate were recorded twice using tail-cuff plethysmography (Systola, Neurobotics, Russia). The first recording served to familiarize the animals with the procedure and the second one provided the pre-pregnancy values of hemodynamic parameters. The recordings were also performed on GD 5-7, GD15, and GD20. At every time point, the measurements were performed at least 5 times for each rat, the obtained values were averaged. On GD18-19, blood samples were taken from the incision of the tail tip (about 300 μL) from the females to determine the content of NO metabolites.

Rat pups of both sexes born to pregnant females were decapitated on postnatal days 1–2 (humane endpoint due to high incidence of limb defects). Trunk blood was collected, the heart and both kidneys were weighed and the aorta was isolated. For functional studies, two 2 mm long ring segments were cut from the thoracic aorta between the aortic arch and the diaphragm. Tissue samples collected for gene expression included the thoracic aorta and a proximal part of the abdominal aorta, these samples were placed in RNA-later (Qiagen) and stored at −20 °C until further analysis.

### 4.2. Measurement of NO Metabolites in Blood Serum

To obtain serum, blood samples were kept at room temperature for 20 min and +4 °C for 40 min, then centrifuged at 4300× *g* for 15 min. Serum was collected and stored at −20 °C until further analysis. Determination of NO metabolites content was carried out using the Griess method after deproteinization of the samples, extraction of lipids, and reduction of NO_3_− to NO_2_− using VCl_3_ simultaneously with the Griess reaction [33]. Optical density was measured spectrophotometrically at 540 nm using Multiskan EX (Thermo Electron Corporation, Langenselbold, Germany).

### 4.3. Determination of the Relative Content of mRNA in the Aorta of Rat Pups

RNA extraction was performed using an RNA isolation kit (Evrogen) according to the manufacturer’s protocol immediately after tissue homogenization in RLT buffer (Qiagen). RNA samples were treated with DNase I (Fermentas, 1000 U/mL) and reverse transcribed for cDNA synthesis (Evrogen, according to the manufacturer’s instructions). Quantitative PCR was performed in a Rotor-Gene 6000 instrument (Corbett Research) using a reagent kit containing the SYBR Green I intercalating dye (Evrogen). The sequences of primers used are listed in Table 3.

GAPDH and RPLP0 were used as reference genes. The mRNA content in the samples was calculated as E^−Ct^, where E is the primer efficiency and Ct is the cycle number at which the product accumulation curve crosses the threshold fluorescence level. The obtained values were normalized to the geometric mean for two reference genes in the same sample. Then the data were averaged within each experimental group and the average value in the control group was taken as 100%.

### 4.4. Functional Experiments on the Isolated Aorta

Aortic ring segments were mounted on steel wires in a multichannel myograph (620M, DMT, Denmark) for isometric force recording. The signal was digitized at a frequency of 10 Hz using an analog-to-digital converter (E14-140, L CARD, Russia); registration was carried out using the PowerGraph 3.3 software (DISoft, Russia). Throughout the experiment, solutions contained in myograph chambers were continuously aerated with carbogen (95% O_2_ + 5% CO_2_), to oxygenate and maintain pH 7.4.

After heating to 37 °C, the optimal passive stretch of the preparation was determined [34] in a calcium-free solution (composition: NaCl—120 mM, NaHCO_3_—26 mM, KCl—4.5 mM, MgSO_4_—1 mM, NaH_2_PO_4_—1.2 mM, D-glucose—5.5 mM, EGTA—0.1 mM, HEPES—5 mM) in the presence of 1 μM NO donor DEA/NO. The preparations were stretched step by step and the force (F) corresponding to the given stretching of the preparation was recorded for 2 min at each step. From this value of the force, the passive tension T=F/2L was calculated, where L is the length of the aortic segment. Using the Laplace equation, the transmural pressure P=T/R=T/(IC/2π) was calculated, where R is the inner radius of the vessel, IC is the internal circumference of the preparation corresponding to the radius R. The internal circumference of the preparation at each step of its stretching was calculated as IC=40×π+2×40+2a, where 40 µm is the diameter of the steel wires on which the preparation was mounted, and a is the distance between the wires. Using the obtained values, the relationship of the inner radius on pressure was plotted and approximated by the equation $P=P_{0}\times \exp\left(K\times IC\right)$, where P_0_ is the pressure corresponding to the adjacent position of the wires, and K is a constant. Using the constant K, the aortic diameter was calculated in the pressure range from 20 to 120 mm Hg. The inner diameter of the vessel corresponding to a pressure of 100 mm Hg (d_100_) was calculated as well.

Further, the aortic rings were set to the optimal stretch corresponding to 0.9d_100_ and placed in a solution of the following composition: NaCl—120 mM, NaHCO_3_—26 mM, KCl—4.5 mM, MgSO_4_—1 mM, NaH_2_PO_4_—1.2 mM, D-glucose—5.5 mM, EDTA—0.025 mM, HEPES—5 mM, and 5–10 min later the cumulative addition of increasing concentrations of Ca^2+^ was carried out into the myograph chamber in the concentration range from 10 μM to 3 mM (the exposure to each concentration lasted for 3 min). To determine the maximum contractile response, the preparations were additionally stimulated with 60 mM KCl. After that, the preparations were washed with a solution of the following composition: NaCl—120 mM, NaHCO_3_—26 mM, KCl—4.5 mM, CaCl_2_—1.6 mM, MgSO_4_—1 mM, NaH_2_PO_4_—1.2 mM, D-glucose—5, 5 mM, EDTA—0.025 mM, HEPES—5 mM. This solution was used during the further experiment.

Relaxation responses of aortic segments were studied against the level of spontaneous precontraction which approximated the level of the maximal contractile response (Figure 2a). Relaxation responses to acetylcholine and the role of NO in this response were studied using two adjacent segments of the same aorta. The NO synthase inhibitor L-NNA (100 μM) was added to one segment, and the same volume of solvent (H_2_O, 50 μL) was added to the other. Twenty minutes later, basal tone values recorded in the absence and the presence of L-NNA were 88.5 ± 3.2% and 94.4 ± 1.1% in the control group and 81.8 ± 2.3% and 83.6 ± 2.1% in the L-NAME group (no statistically significant effects of either group or L-NNA were observed using two-way ANOVA with Sidak posthoc test). Then the concentration-response relationship to acetylcholine was carried out for both segments (concentration range 10 nM—10 μM, the duration of each concentration action was 2 min). After washing from acetylcholine, 100 μM L-NNA was re-added to the previously L-NNA-treated segment and 20 min later the concentration-response relationship on DEA/NO was carried out (concentration range 10 nM—10 μM, the duration of each concentration action was 2 min). Basal tone values before application of the first DEA-NO concentration were 69.5 ± 3.6% in the control group and 78.0 ± 2.9% in the L-NAME group (*p* > 0.05 unpaired Student’s *t*-test). At the end of the experiment, 2 mM EGTA was added to all preparations to determine the level of force that corresponded to the complete relaxation of smooth muscle (the level of “passive” force).

During data analysis, the value of the “passive” force was subtracted from all force values recorded during the experiment. The obtained values of active force were expressed as a percentage of the maximum force of contraction (for contractile responses to Ca^2+^) or a percentage of the precontraction level (for relaxation responses to acetylcholine or DEA/NO). For the reactions of the aorta to acetylcholine, the area above the relaxation curve was calculated using the GraphPad Prism 7.0 software (San Diego, CA, USA).

### 4.5. Statistical Analysis

Statistics were calculated in the GraphPad Prism 7.0 software. The normality of data distribution was checked using the Shapiro-Wilk normality test. Between-group differences were assessed using two-way ANOVA followed by Tukey or Sidak’s posthoc analysis (if needed), Student’s *t*-test, or Mann-Whitney test. Differences were considered statistically significant at *p* < 0.05, *n* is the number of rats in the group.

## 5. Conclusions

To conclude, our novel results based on in vivo model of maternal NO-deficiency demonstrate that NO is important for smooth muscle cell-specific gene expression during early ontogenesis. Consequently, a high level of NO production in early ontogenesis observed previously by our and other groups in vascular endothelium [1] is important not only for maintaining a low level of blood pressure in still-immature vessels but is also necessary for phenotypic modulation of vascular smooth muscle cells, their transition to a mature contractile phenotype.

## Figures and Tables

**Table 1:** Bodyweight (BW), organ weights as well as concentrations of NO metabolites in serum of 1-day-old rat pups from control and L-NAME groups.

Parameters	Control	L-NAME
BW, g (*n* = 17;20)	6.9 ± 0.5	5.6 ± 0.7 *
Heart weight/BW, % (*n* = 17;20)	0.46 ± 0.03	0.51 ± 0.04 *
Kidney (both) weight/BW, % (*n* = 17;20)	0.86 ± 0.10	0.87 ± 0.12
NOx, µM (*n* = 10;10)	39 ± 11	14 ± 8 *

The day of birth was considered as postnatal day 0; *n*—number of rat pups. Data are presented as mean and S.E.M. * *p* < 0.05 Unpaired Student’s *t*-test).

**Table 2:** Aortic characteristics of 1–2-day-old rat pups from control and L-NAME groups.

Parameters	Control (*n* = 6)	L-NAME (*n* = 14)
Inner diameter d_100_, µm	854 ± 39	765 ± 119 *
Maximum active tension, mN/mm	1.07 ± 0.18	1.14 ± 0.24

Data are presented as mean and S.E.M. * *p* < 0.05 (Unpaired Student’s *t*-test).

**Table 3:** Gene-specific primers used in qPCR.

Protein	Gene	Forward	Reverse
eNOS	*Nos3*	GGATTCTGGCAAGACCGATTAC	GGTGAGGACTTGTCCAAACACT
nNOS	*Nos1*	GCCATCCAGCGCATAATGACCCAG	GAGGGTGACTCCAAAGATGTCCTC
iNOS	*Nos2*	AGGCTTGGGTCTTGTTAGCCTAGT	ATTCTGTGCAGTCCCAGTGAGGAA
Arginase-2	*Arg2*	CCAGCCTAGCAGTGGATGTGA	CTCTGGAATGCTGTCGTGAA
SM-MHC	*Myh11*	TTTGCCATTGAGGCCTTAG	GTTCACACGGCTGAGAATCCA
α-Actin	*Acta2*	CCTGACCCTGAAGTATCCGA	CATCTCCAGAGTCCAGCACA
SM22α	*Tagln*	TTCTGCCTCAACATGGCCAAC	CACCTTCACTGGCTTGGATC
Cav1.2	*Cacna1c*	CATCTCCATCACCTTCTTCC	AAATACCTGCATCCCAATCAC
RyR2	*Ryr2*	GAGAGCCCGGAAGCTCTGAA	GGCAACTCCATGGCACACAC
SERCA2A	*Atp2a2*	TGAATCTGACCCAGTGGCTGA	ACTCCAGTATTGCAGGCTCCA
GAPDH	*Gapdh*	CACCAGCATCACCCCATTT	CCATCAAGGACCCCTTCATT
RPLP0	*Rplp0*	AGGGTCCTGGCTTTGTCTGTGG	AGCTGCAGGAGCAGCAGTGG

## Data Availability

All data generated during this study are available from the corresponding author on reasonable request.
